# Genetic Characterization and Comparative Genome Analysis of *Brucella melitensis* Isolates from India

**DOI:** 10.1155/2016/3034756

**Published:** 2016-07-25

**Authors:** Sarwar Azam, Sashi Bhushan Rao, Padmaja Jakka, Veera NarasimhaRao, Bindu Bhargavi, Vivek Kumar Gupta, Girish Radhakrishnan

**Affiliations:** ^1^National Institute of Animal Biotechnology, Hyderabad 500049, India; ^2^Ella Foundation, Genome Valley, Turkapally, Shameerpet, Hyderabad 500078, India; ^3^Central Institute for Research on Goats, Makhdoom, Mathura 281122, India

## Abstract

Brucellosis is the most frequent zoonotic disease worldwide, with over 500,000 new human infections every year.* Brucella melitensis*, the most virulent species in humans, primarily affects goats and the zoonotic transmission occurs by ingestion of unpasteurized milk products or through direct contact with fetal tissues. Brucellosis is endemic in India but no information is available on population structure and genetic diversity of* Brucella* spp. in India. We performed multilocus sequence typing of four* B*.* melitensis* strains isolated from naturally infected goats from India. For more detailed genetic characterization, we carried out whole genome sequencing and comparative genome analysis of one of the* B*.* melitensis* isolates, Bm IND1. Genome analysis identified 141 unique SNPs, 78 VNTRs, 51 Indels, and 2 putative prophage integrations in the Bm IND1 genome. Our data may help to develop improved epidemiological typing tools and efficient preventive strategies to control brucellosis.

## 1. Introduction

Brucellosis is a worldwide zoonotic disease that accounts for huge loses to the livestock sector and poses a serious threat to public health. The disease is caused by bacteria of the genus* Brucella*, a member of the *α*-2 Proteobacteria [1]. Brucellae are Gram-negative, facultative, intracellular bacteria that can infect a wide range of domestic and wild animals as well as humans. Six classical species were initially recognized within the genus* Brucella,* namely,* B*.* abortus*,* B*.* melitensis*,* B*.* suis*,* B*.* ovis*,* B*.* canis*, and* B*.* neotomae* [2].* Brucella* invades and replicates in professional phagocytic cells such as macrophages and dendritic cells as well as nonprofessional phagocytes such as trophoblasts [3–5].* Brucella* mechanisms and virulence factors that mediate invasion and intracellular persistence have been poorly characterized.

Brucellosis remains endemic and is a reemerging disease in many regions of the world including Latin America, Middle East, Africa, Central Asia, and the Mediterranean basin that affects human health and animal productivity. The disease leads to a significant detrimental effect on local economies resulting in the perpetuation of poverty [6]. Brucellosis is a serious veterinary and public health problem in India and the disease is reported in cattle, buffalo, sheep, goats, pigs, camel, dogs, and humans [7]. Brucellosis is an endemic disease in India and the country experienced a sharply increasing rate of human brucellosis in recent years, and the species of main concern is* B*.* melitensis*.

*B*.* melitensis*, the most virulent species in humans, primarily affects goats and the zoonotic transmission occurs by ingestion of unpasteurized milk products or through direct contact with fetal tissues. Genetic diversity and population structure of* Brucella* spp. remain unknown in India. Multilocus sequence typing (MLST) has been considered as the robust tool for dissecting genetic diversity and population structure within the bacterial species. The established MLST schema for* Brucella* spp. employed nine highly distinct genomic loci [8]. However, MLST resolution is limited and often fails to differentiate closely related strains. With the advent of next generation sequencing, whole genome offers new opportunities to analyze the genetic diversity among the groups [9]. Genome sequencing and analysis of* Brucella* spp. from diverse hosts and geographical regions have been reported [9]. Until now, 61 genomes of* B*.* melitensis* have been sequenced and made available in the GenBank.* B*.* melitensis* contains more than 3000 genes that are distributed over two circular chromosomes. These sequenced species serve as vast resources for comparative genomics and understanding the evolutionary history. Comparative genomics will provide insights into the virulence mechanisms of the* Brucella* spp. such as novel genomic islands and integration of prophages and SNPs that regulate the expression of certain genes or affect the function of important virulence-associated proteins. One of the previous studies demonstrated conservation of genes and genomic islands across the different* Brucella* spp. [10]. In a recent study, the evolutionary relationship of* B*.* melitensis* on the basis of whole genome SNPs revealed spatial clustering of* B*.* melitensis* isolates into five genotypes [11]. All the Asian isolates of* B*.* melitensis* clustered into genotype II, whereas the isolates from Europe and America clustered into genotypes IV and V, respectively.

We performed genotyping by MLST on* B*.* melitensis* strains isolated from naturally infected goats. To perform detailed genetic characterization and comparative genome analysis, we performed whole genome sequencing of one of the strains,* B*.* melitensis* IND1, using the Illumina platform. The analysis revealed the extent of genetic variation of* B*.* melitensis* IND1 in comparison to* B*.* melitensis* isolates from other geographical locations. Data generated from our studies may help to develop new diagnostic assays based on stable markers such as SNPs (Single Nucleotide Polymorphisms) and Variable Number of Tandem Repeats** (**VNTRs) for molecular epidemiological studies. Identification of SNPs, Indels, and novel phage integration sites will provide insights into the virulence mechanisms of this stealthy pathogen which could ultimately lead to the development of novel therapeutic and preventive strategies to control brucellosis.

## 2. Materials and Methods

### 2.1. Isolation of* B. melitensis*

For isolation of* Brucella*, materials from four different sources are listed in the Supplementary Table  1 (in Supplementary Material available online at http://dx.doi.org/10.1155/2016/3034756). Samples were inoculated on sterile plates of* Brucella* selective agar containing Hemin and Vitamin K_1_ media (Hi Media, India) and incubated at 37°C for 48 hours. The plates were observed at every 24 hours for the development of growth. After obtaining the growth, the colonies suspected for* Brucella* on the basis of cultural characteristics were selected and streaked again on plates containing Brucella selective agar with Hemin and Vitamin K_1_ and incubated at 37°C for 2 days to obtain the pure culture.

### 2.2. Biotyping of* Brucella* Isolates

Cultures showing typical* Brucella* characteristics were subjected to biotyping techniques such as H_2_S production, growth in the presence of thionin, and basic fuchsin (10–40 *μ*g/mL) dye incorporated into tryptic soy agar at different concentrations and CO_2_ requirement immediately after the primary isolation as described [12]. Lead acetate strips were used to identify the production of H_2_S during growth, and the growth was evaluated on media containing streptomycin (2.5 *μ*g/mL) to discriminate the isolates from vaccine strain Rev1 as described [13].

Genomic DNA of all five strains was isolated using the Wizard Genomic DNA Purification Kit (Promega, USA). Isolated DNA was used for polymerase chain reactions to amplify 16S rRNA and the Omp 31 gene for the confirmatory identification of* Brucella melitensis* using the Taq PCR master mix kit (Qiagen). 16S rRNA is specific to the genus* Brucella* while Omp 31 is a species-specific gene to* Brucella melitensis* [14–16].

### 2.3. MLST Analysis of* B. melitensis* Isolates

For MLST analysis, 4,396 nucleotide sequences spanning nine genomic fragments from* Brucella* were selected as described [8]. Of the nine loci, seven belong to classical housekeeping genes, one locus derived from the outer membrane protein 25 gene, and one from an intergenic region. Genomic DNA was isolated from Bm IND isolates using the Wizard Genomic DNA Purification Kit (Promega). Genomic fragments were amplified by PCR using the following cycling parameters: 94°C for 2 min, 35 cycles of 94°C for 30 sec, 53°C for 30 sec, and 72°C for 1 min and 72°C for 5 min. Primers used for MLST analysis are listed in the Additional file 13. An aliquot of the PCR amplicons was analyzed by 1% agarose gel and photographed. Remaining PCR products were purified and subjected to Sanger sequencing using the forward and reverse primers that were used for PCR amplifications. Editing of the sequences and generation of contigs from forward and reverse sequences was performed using Lasergene 8 software (DNA Star, USA). To perform the phylogenetic analysis, nine genomic fragments mentioned above were fetched from representative* Brucella* species and the loci were amplified* in silico* using the MLST primers. All the sequences were concatenated to identify the allelic profile with the help of Brucellabase [17] and the concatenated sequences were subjected to multiple sequence alignment using MAFFT version 7.123b [18]. Phylogenetic analyses were performed with RAxML version 8.1.2 [19] using GTRGAMMA model of evolution. The phylogram was visualized using Dendroscope version 3 [20].

### 2.4. Genome Sequencing, Assembly, and Annotations

The complete genome of* Brucella melitensis* IND1 (Bm IND1) was sequenced using Illumina technology. The sequenced data have been deposited at DDBJ/EMBL/GenBank under the accession number JMKL00000000 [21]. Paired end data generated were filtered for low quality reads using in-house script. After preprocessing, high quality data were used to make a scaffold level assembly using the SOAPdenovo version 2.01 assembler [22]. Scaffolds were further mapped with raw reads using bowtie2 version 2.2.4 [23], and coverage at each base was calculated using SAMtools version 0.1.19 [24]. Graphs for coverage analysis were plotted using GNUplot version 4.6 (http://www.gnuplot.info/).

Completely sequenced genomes of* B*.* melitensis* such as* B*.* melitensis* 16M,* B*.* melitensis* M28,* B*.* melitensis* ATCC23457, and* B*.* melitensis* NI were initially considered as candidates for the template to construct the chromosomal assembly of Bm IND1. The raw data of Bm IND1 were aligned onto all the genomes using bowtie2 and the SNPs were identified using SAMtools [24], BCFtools, and VCFtools (http://vcftools.sourceforge.net/). Highly confident SNPs were filtered out using scripts from ISMU pipeline [25] based on the criteria that the raw read depth is greater than 10 and there is no reference base in the alignment. Finally,* B*.* melitensis* ATCC 23457 that showed the minimum number of SNPs with Bm IND1 was used as the template for chromosome level assembly. Abacas version 1.3.1 [26] was used to assemble Bm IND1 scaffolds into two chromosomes. Bm IND1 chromosomal level assembly was further manually curated using the BLAST output of Bm IND1 scaffolds with the* B*.* melitensis* ATCC 23457 genome. We compared the syntenic relationships between Bm IND1,* B*.* melitensis* ATCC 23457, and* B*.* melitensis* 16M using Mauve version 2.3.1 [27]. The structural and functional annotations of Bm IND1 genome were carried out by RAST server [28].

### 2.5. Whole Genome Phylogeny

Genomes of* B*.* melitensis* isolates were downloaded from GenBank (ftp://ftp.ncbi.nih.gov/genomes/genbank/bacteria/Brucella_melitensis/). First, we assessed the completeness of the assembly and annotations of each sequenced genome and filtered out the incomplete ones. To assess the core genome and single copy orthologs, Orthomcl v.1.4 [29] was used with default parameters. We used 2319 single copy orthologs to construct a maximum-likelihood tree following the approach of Wattam et al. [10]. MAFFT version 7.123b [18] was used to align sequences from each gene family independently. All the alignments were further processed and concatenated using Gblocks version 0.91b [30]. RAxML version 8.1.21 [19] was used to generate a tree for all the dataset using the PROTGAMMALG model of evolution. The tree was visualized using Dendroscope version 3 [20].

### 2.6. Detection of Prophages

The genome was searched for prophage sequences and phage attachment site using PHAST (phage search tool), available at http://phast.wishartlab.com/ [31].

### 2.7. Identification of Variable Number of Tandem Repeats (VNTRs)

Tandem repeats in each chromosomes of Bm IND1 were identified using Tandem repeat finder [32]. A precompiled Tandem repeat finder version 4.07b was downloaded from http://tandem.bu.edu/trf/trf.download.html and run on Linux (64-bit) platform using the parameter of a minimum alignment score of 80.

### 2.8. Identification of SNPs

We downloaded the genome sequences of* B*.* melitensis* 16M,* B*.* melitensis* M28,* B*.* melitensis* ATCC 23457,* B*.* melitensis* M5-90,* B*.* melitensis* NI,* B*.* melitensis* Ether, and* B*.* abortus* 2308 for SNP analysis. We considered only the completely assembled genomes for analysis of SNPs. We established a pipeline for finding SNPs between two reference sequences using Nucmer and show-snps program from the Mummer3 package [33]. Show-snps provide SNPs derived only from uniquely aligned regions. SNPs were extracted from each strain against Bm IND1 and the data were further annotated using SnpEff [34] to predict SNP effects in the genome.

### 2.9. Indels Analysis

To find insertions and deletions in the coding region, VCF files generated against* B*.* melitensis* ATCC 23457 using Bm IND1 reads for template genome selection were annotated with SnpEff. Indels in the coding regions and their corresponding functions were extracted from* B*.* melitensis* ATCC 23457 using in-house Perl script.

## 3. Results and Discussion

### 3.1. Isolation and Genotyping of* B. melitensis* IND Strains

**Figure 1:**
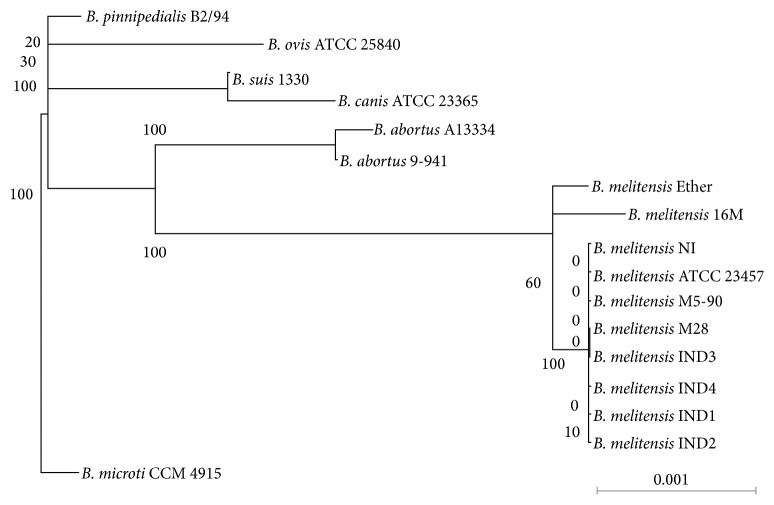
Phylogenetic tree based on MLST analysis. Seven housekeeping genes and two loci from outer membrane protein 25 and an intergenic region, respectively, were used for MLST analysis. Bootstrap percentages retrieved in 100 replications are shown at the nodes.

We isolated four strains of* B*.* melitensis* from naturally infected goat followed by MLST analysis to understand the genetic diversity among the* B*.* melitensis* IND strains. To perform MLST analysis, we amplified nine loci that included seven housekeeping genes, one locus from the outer membrane protein 25 (omp25), and one locus from an intergenic region (Supplementary Figure  1). The inclusion of loci from omp25 and intergenic region was reported to have provided more discriminatory power in the phylogenetic analysis [8]. Subsequently, we compared the allelic profiles of the four* B*.* melitensis* IND isolates with each other and with other reported* Brucella* species. All the* B*.* melitensis* IND isolates displayed identical sequences with an allelic profile of 3-2-3-2-1-5-3-8-2, which belong to Sequence Typing- (ST-) 8. The phylogram was rooted with* B*.* microti* and in the phylogram all the* B*.* melitensis* isolates clustered into one lineage (Figure 1). Bm IND strains grouped with other Asian strains, that is,* B*.* melitensis* M28,* B*.* melitensis* M5-90, and* B*.* melitensis* NI, whereas* B*.* melitensis* Ether and* B*.* melitensis* 16M branched separately. This was anticipated as all the Asian strains belong to ST-8 and* B*.* melitensis* Ether and* B*.* melitensis* 16M falls into ST-9 and ST-7, respectively. In fact, the support value is very low for the branches of Asian isolates in the phylogram owing to the same allelic profile (ST-8) of MLST loci that were considered for the phylogenetic analysis. As expected,* B*.* suis* and* B*.* ovis* clustered into different clades in the phylogram (Figure 1). The analysis indicates that* B*.* melitensis* with ST-8 is prevalent in Asia.

### 3.2. Whole Genome Sequencing, Assembly, and Annotation of* B*.* melitensis* Strain Bm IND1

We performed the whole genome sequencing of Bm IND1 to analyze the genetic divergence and genomic features in detail. The raw data generated using the Illumina sequencing platform were assembled using SOAPdenovo. This provided 102 contigs that were further assembled into 29 scaffolds (Table 1). Mapping reads onto them further validated these scaffolds. On average, each scaffold base was covered more than 100 times (100x); however, some scaffolds like scaffolds 9, 14, 18, 20, and 22 have shown high depth of coverage (Supplementary Figure  2). This is likely due to the presence of duplicated or repeat regions of the genome.

To generate chromosome level assembly, the scaffolds were assigned to two* Brucella* chromosomes with proper order and orientation. Generally, a reference genome of a closely related species is used as the template to align and order the scaffolds. To achieve this, we considered genomes of* B*.* melitensis* 16M reported from USA,* B*.* melitensis* NI from Mongolia,* B*.* melitensis* Ether from Italy,* B*.* melitensis* ATCC 23457 from India, and the* B*.* melitensis* M28 from China. All these genomes are completely sequenced and well annotated and the genome assembly of few has previously been used as reference genomes for other strains [35, 36]. We aligned the raw data of Bm IND1 on the genomes of above* B*.* melitensis* strains using bowtie2 and identified the SNPs (Supplementary Table  2). Since* B*.* melitensis* ATCC 23457 displayed the least number of SNPs, which indicates minimum genetic divergence, we selected the genome of this strain as the reference genome for chromosomal assembly of Bm IND1.* B*.* melitensis* IND1 and* B*.* melitensis* ATCC 23457 were isolated from India; however, they belong to different biovars.

**Figure 2:**
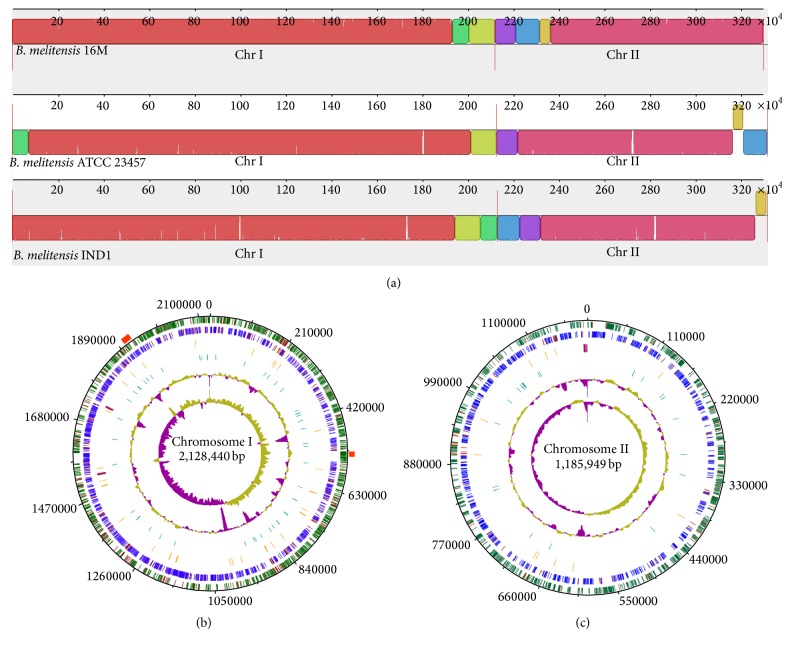
(a) Alignment of* B*.* melitensis* 16M,* B*.* melitensis* ATCC 23457 and* B*.* melitensis* IND1 genome. Mauve alignment shows the synteny regions between the three strains. Bm IND1 and* B*.* melitensis* ATCC 23457 aligned well with each other; however, a segment (olive color block) on Chromosome II of Bm IND1 is in reverse orientation in* B*.* melitensis* 16M. ((b) and (c)) Circular representation of* B*.* melitensis* IND1 Chromosome I (b) and Chromosome II (c). Chromosomal coordinates are indicated on outer most circle. Circles are represented from outer to inner as circle 1, CDS on the positive strand (green for annotated, red for hypothetical); circle 2, CDS on negative strand (blue for annotated, red for hypothetical); circle 3, RNA genes (orange for tRNA and purple for rRNA); circle 4, VNTRS (turquoise); circle 5, GC content (olive for positive and purple for negative); circle 6, GC skew (olive for positive and purple for negative). Red blocks above circle 1 represent phage integration site in Chromosome I.

Bm IND1 scaffolds were assembled into two chromosomes using* B*.* melitensis* ATCC 23457 as the template. Total number of scaffolds assigned on Chromosomes I and II are 23 and 6, respectively. Subsequently, assembly was manually curated with a focus on those scaffolds that showed higher physical coverage to fix the duplications. We observed the duplications of scaffolds 20, 22, 27, 28, and 29 on Chromosome I and scaffold 26 on Chromosome II with respect to the* B*.* melitensis* ATCC 23457 genome. These manually placed scaffolds were in concordance with observed physical coverage. However, no duplication in other scaffolds was observed, especially scaffolds 9 and 14 that showed high coverage (Supplementary Figure  2(b)). We assume that the higher coverage in these scaffolds may be due to internal repeats or sequencing bias. Next, we aligned the genomes of* B*.* melitensis* ATCC 23457,* B*.* melitensis* 16M, and Bm IND1 and observed for macrolevel synteny and large genomic rearrangements. In fact, they were highly syntenic with each other except for one segment of* B*.* melitensis* 16M on Chromosome II which was in reverse orientation in* B*.* melitensis* ATCC 23457 and Bm IND1 (Figure 2(a)). All general features of the genome are summarized in Figures 2(b) and 2(c).

**Figure 3:**
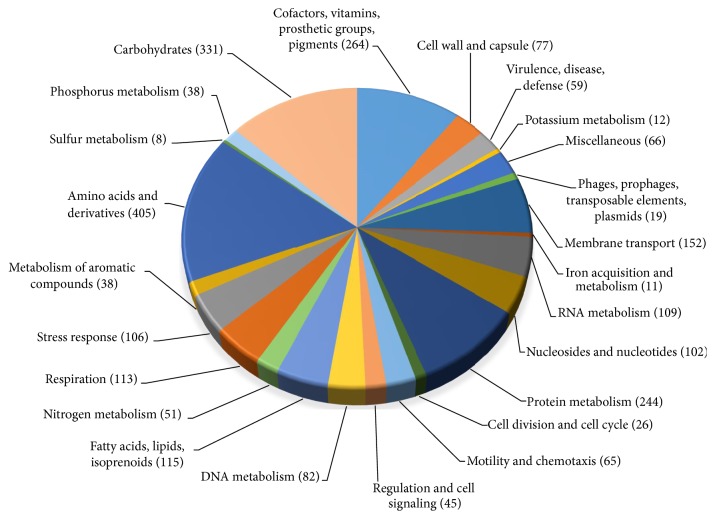
Distribution of subsystem category for* B*.* melitensis* IND1. Bm IND1 genome sequence was annotated using Rapid Annotation System Technology server. Features of each subsystem and their coverage are summarized in the pie chart.

We annotated the genome of* B*.* melitensis* Bm IND1 using Rapid Annotations with Subsystems Technology (RAST) to obtain the coding and noncoding genes [28]. A total of 55 tRNA, 12 rRNA, and 3191 protein coding genes with an average CDS length of 874 bp were annotated (Table 2). In addition, RAST annotates the genomic structures and assigns their functions on the basis of presence of subsystems in the genome. This makes functional annotation of genes more accurate than simply assigning the functions on the basis of sequence similarity of known genes. Functions of 2562 genes were assigned while 629 genes were annotated as hypothetical (Table 2). A total of 1649 genes were assigned for different subsystems where maximum number (405) was assigned for metabolism of amino acids followed by carbohydrate metabolism (331) (Figure 3).

#### 3.2.1. Whole Genome Phylogeny

**Figure 4:**
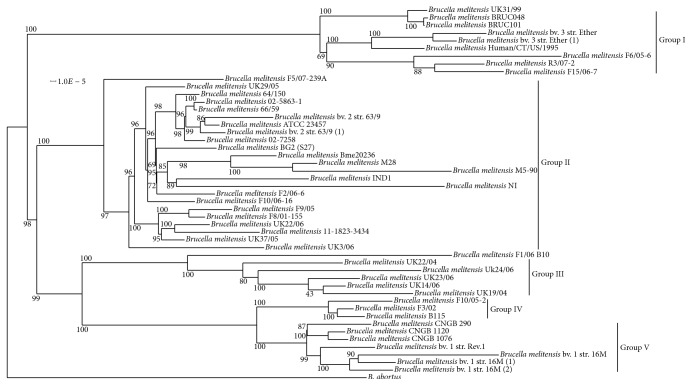
Phylogenetic tree showing relationship between* B. melitensis* IND1 and other* B. melitensis* strains. Maximum likelihood tree of 49 whole genome sequenced* B. melitensis* strains, inferred from concatenated, partitioned alignment of 2319 core genes using RAxML. Support values of branches are calculated from 100 bootstrap replicates and the branch length is proportional to the number of substitutions per site.* B. abortus* 2308 has been used as outgroup species.

Determining the phylogenetic relationship in a bacterial population is essential to understand the population structure, evolutionary history, and host relationship and to develop diagnostic assays for molecular epidemiological studies [9]. To perform comparative phylogenomics, we downloaded all the currently available* B*.* melitensis* genomes (59 genomes) from GenBank and considered* B*.* abortus* 2308 as the outgroup species. We evaluated the completeness of assembly and annotation of the genomes by assessing the number of orthologous genes, which are highly conserved among the strains. Any* B*.* melitensis* strain showing less number of orthologous genes than the number of orthologous genes present in* B*.* abortus* with respect to* B*.* melitensis* were ignored for the downstream phylogenomic studies. This facilitates more accurate core genome estimation and identification of single copy orthologs present in the species, which improves resolution of the phylogenetic tree. Therefore, we ignored the genomes of 12* B*.* melitensis* strains and considered the entire repertoire of coding genes of 48* B*.* melitensis* strains including Bm IND1 for the analysis (Supplementary Table  3). A total of 151361 genes of* B*.* melitensis* strains were clustered in 3800 gene families, of which 25124 gene families present in all the 48 strains. However, 2461 genes only showed exact single copy orthology in each strain that could be considered as the core genome of* B*.* melitensis* clade. The core genome includes 73–82% of genes from each of the* B*.* melitensis* strain. Wattam et al. [10] reported that 2,285 core genes are present in the* Brucella* genus by analyzing the genomes of 40* Brucella* species. Conceivably, the core genome of* B*.* melitensis* clade was higher than the total number of core genes present across the* Brucella* genus. We used* B*.* abortus* 2308 as the outgroup for whole genome phylogeny that increased the total cluster of genes to 3829. After including the* B*.* abortus* strain, the total number of single copy orthologs decreased to 2319 genes. In the whole genome phylogram, Bm IND1 clustered with other Asian isolates of* B*.* melitensis* as observed in the MLST analysis (Figure 4). Bm IND1 grouped with* B*.* melitensis* NI which was originated from Mongolia and both the strains belong to biovar 3 (Figure 4). The phylogenetic relationship established here is in agreement with the earlier reports [10, 11]. Tan et al. [11] performed a comparative whole genome SNP analysis of* B*.* melitensis* strains from around the world and reported clustering of* B*.* melitensis* isolates into five genotypes. In agreement with this observation, our phylogenomic studies also revealed the clustering of* B*.* melitensis* isolates into five groups where Group 1 formed the earliest diverging clade. Group II represents most of the Asian isolates of* B*.* melitensis* including Bm IND1. Parallel to Group II, another lineage evolved which further branched into groups III, IV, and V. Group III represents isolates from Africa and groups IV and V constitute isolates from Europe and America, respectively.

#### 3.2.2. Prophages

**Figure 5:**
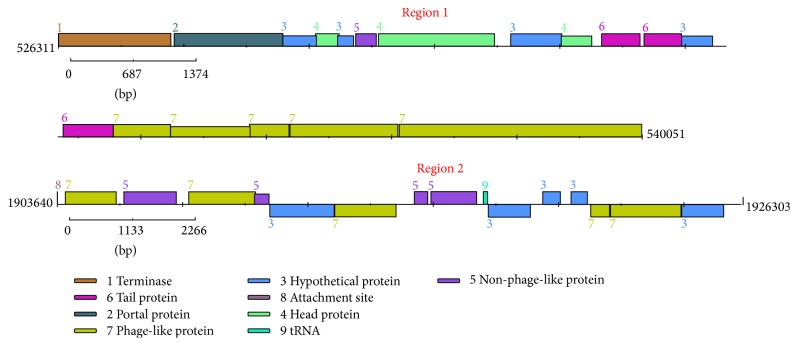
Genomic organization of two putative phage like regions. Scales are described below the chromosomal region and legends are described at the bottom.

Prophages that are integrated into the genome of bacteria can contribute many biological properties to their bacterial hosts such as virulence, biosynthesis, and secretion of toxins, genomic divergence, and evolution [37]. We analyzed the genome of Bm IND1 for prophages using PHAST that was designed to identify and annotate prophage sequences in bacterial genomes [31]. The analysis identified 2 putative prophage integrations in Chromosome I of Bm IND1 (Table 3 and Figure 5). Region 1 is composed of a fragment of 13.7 kb size that encoded 18 genes, out of which 14 genes were phage specific and 4 genes were bacteria specific. Region II is composed of 22.6 kb with 14 genes where 8 genes were phage specific and remaining 6 genes belonged to* Brucella*. Notably, region 1 is considered as intact prophage upstream of QseB locus and the RAST server could identify the genes in this region. Region II is predicted as incomplete prophage but flanked with attachment sites. Region I is present in the Chinese isolate of* B*.* melitensis* M28 also. We identified two putative phage integrations in Chromosome I of* B*.* melitensis* 16M genome, which did not show any similarity to that of Bm IND1 or* B*.* melitensis* M28. The analyses clearly indicate that the prophage integration events contribute to the genetic diversity of* B*.* melitensis*.

#### 3.2.3. VNTRs

VNTRs play an important role in evolution, gene regulation, genome structure, antigenic variation and virulence [38–40]. Mutations in VNTRs produce a wide range of allelic diversity and VNTRs are considered as a powerful technique in molecular typing of bacterial species [41–43]. We have analysed the genome of Bm IND1 and identified 78 VNTRs with DNA motif size ranges from 8 to 30 bps and the copy number ranges from 1.9 to 10.4 (Supplementary Table  4). The data generated in our analysis could be used for developing rapid diagnostic assays for high-resolution molecular epidemiological and clinical studies.

#### 3.2.4. SNPs

**Figure 6:**
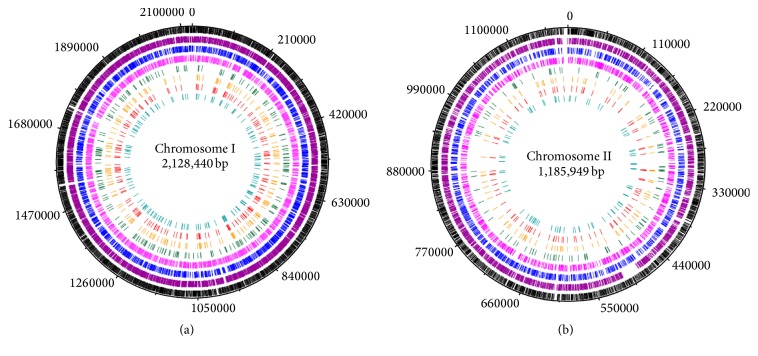
Circular representation of identified Single Nucleotide Polymorphisms in* B*.* melitensis* IND1 Chromosome I (a) and Chromosome II (b). Chromosomal coordinates are indicated on outer most circle. From outer to inner, circles represented as circle 1, CDS (black); circle 2, SNPs against* B*.* abortus* 2308 (purple); circle 3, SNPs against* B*.* melitensis* Ether (blue); circle 4, SNPs against* B*.* melitensis* 16M (pink); circle 5, SNPs against* B*.* melitensis* NI (green); circle 6, SNPs against* B*.* melitensis* M5-90 (Orange); circle 7, SNPs against* B*.* melitensis* M 28.

SNPs serve as a powerful tool to describe the phylogenetic framework of a species [44]. SNPs data will help to develop novel high-resolution molecular typing techniques for inter- and intraspecies discrimination of pathogenic microorganisms. We compared the genome of Bm IND1 with seven other* B*.* melitensis* strains, that is,* B*.* melitensis* 16M,* B*.* melitensis* M28,* B*.* melitensis* ATCC 23457,* B*.* melitensis* M5-90,* B*.* melitensis* NI,* B*.* melitensis* Ether, and* B*.* abortus* 2308 for SNPs (Table 4 and Figure 6). The highest number of SNPs was detected with* B*.* abortus* as it belonged to a different species (Table 4). Four* B. melitensis* strains that are originated from Asia, namely,* B*.* melitensis* M28 bv1,* B*.* melitensis* M5-90 bv1,* B*.* melitensis* ATCC 23457 bv2, and* B*.* melitensis* NI bv3 exhibited fewer SNPs ranging from 252 to 351 indicating their close genetic relatedness irrespective of their biovars. This observation was in agreement with the reported SNP-based phylogenetic analysis by Tan et al. [11]. However, the SNPs observed with different strains in Additional file 4 were less than the SNPs detected from NGS raw reads for template genome selection. This is because the polymorphisms extracted in these cases were derived from uniquely aligned regions between two genome sequences. Most of the identified SNPs were in the coding regions of the genomes that may be attributed to the high proportion of coding regions in bacteria.

**Figure 7:**
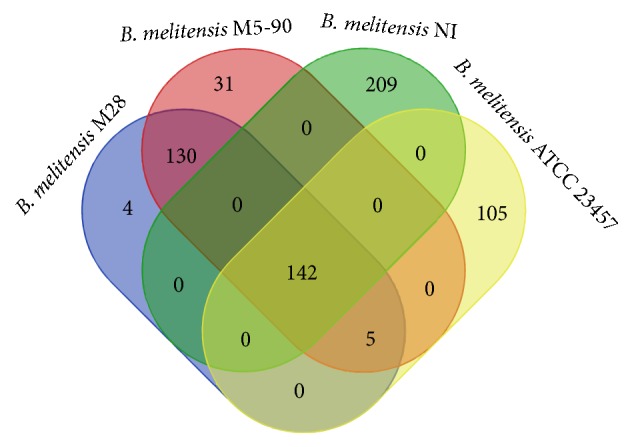
The Venn diagram of SNPs detected in different Asian strains against* B*.* melitensis* IND1.

While analysing the distribution of SNP locus among all Asian strains, 142 SNPs were shared by four strains, namely,* B*.* melitensis* M28,* B*.* melitensis* ATCC 23457,* B*.* melitensis* M5-90, and* B*.* melitensis* NI (Figure 7). Out of 142 SNPs, 141 are shared by all the 7 strains included in the polymorphism analysis (Table 5). Therefore, these 141 unique loci in Bm IND1 could be employed for genotyping and other molecular epidemiological studies.* B*.* abortus* 2308,* B*.* melitensis* Ether, and* B*.* melitensis* 16M shared the maximum number of SNPs (952) against Bm IND1. These 952 loci are conserved in all Asian strains, which may indicate that Asian strains evolved from a common ancestor, and these loci mutated before its differentiation. This finding is in agreement with whole genome phylogenetic analysis where* B*.* melitensis* M28,* B*.* melitensis* M5-90,* B*.* melitensis* NI, and Bm IND1 clustered together as a separate clade (Figure 4). However, 4864 SNP loci are highly specific to* B*.* abortus* 2308, which are not shared by any of the six* B*.* melitensis* strains. These loci with interspecific polymorphisms can differentiate these two species in clinical and epidemiological studies. Also, identified SNPs that are unique to each* B*.* melitensis* strain could be employed for in-depth molecular analysis and development of novel molecular typing tools.

We also categorized the genes containing SNPs based on their functions assigned by RAST server (Supplementary Figure  3). The subsystem category, which has shown the highest proportion of genes containing SNPs, is nitrogen metabolism followed by phosphorous, carbohydrate, and amino acid metabolism. Our findings are in agreement with differential utilization of carbohydrates and amino acids by closely related* Brucella* species and biovars. A biotyping system has recently been developed to discriminate* Brucella* species and biovars based on their differential metabolic activity [45].

#### 3.2.5. Indels

**Figure 8:**
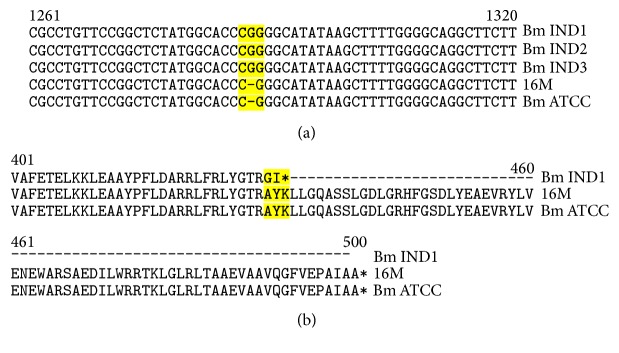
Sequence alignment of 3 terminus regions of glpD gene. (a) Insertion of “G” in the glpD gene (position 1286) of Bm IND strains in comparison to* B*.* melitensis 16M* (16M) and* B*.* melitensis* ATCC23457 (Bm ATCC). (b) Deletion of 69 nucleotides from the C-terminus of glpD of Bm IND1 as a result of the nucleotide insertion.

Indels refers to deletions or insertions of nucleotides in one genome with respect to another, which could be employed as sequence signatures to characterize evolution of diverged organisms [46]. Indels can have a drastic effect on a gene leading to frameshift, truncations, or extensions of an encoded protein. We have identified 51 Indels in the Bm IND1 genome with respect to* B*.* melitensis* ATCC 23457, of which 25 are located in the coding regions (Table 6). One noted INDEL is in the glycerol-3-phosphate dehydrogenase gene (*glpD*) of Bm IND1. Insertion of a guanosine residue in the 3′ end of* glpD* resulted in two amino acid mismatch followed by deletion of 69 amino acids from the C-terminus of this protein. We verified this Indel by PCR amplification and sequencing of corresponding regions from the glpD gene of* B*.* melitensis* IND strains including Bm IND1. Insertion of G was present in the glpD gene of all the four* B*.* melitensis* IND strains with respect to ATCC23457 and 16M (Figure 8). It has been reported that* B*.* melitensis* 16M deficient in glpD was attenuated in human and mouse macrophages [47, 48]. However, our preliminary infection studies did not indicate attenuation of Bm IND1 in mouse peripheral blood mononuclear cells. Our future studies will focus on* in vitro* and* in vivo* infections studies using Bm IND1 to analyse its virulence properties with the objective of developing novel live attenuated vaccines for livestock brucellosis.

## 4. Conclusions

In conclusion, genomic characterization and comparative genome analysis of Bm IND1 revealed genetic structure of* B*.* melitensis* from India as well as from other geographical locations. Comparative genome analysis identified the sources of genetic variation in the form of SNPs, VNTRs, prophages, and Indels. These genetic markers could be employed for developing high-resolution epidemiological typing tools to understand the structure of* Brucella* population and for outbreak analysis. Information on prophage integration events and Indels in the virulence-associated genes will provide important leads for the further experimental characterization of virulence properties of Bm IND1. This may ultimately lead to the development of efficient therapeutic and preventive strategies to control animal and human brucellosis.

## Supplementary Material

Indels identified in the genome of Bm IND1 with respect to *B. melitensis* ATCC 23457. Indels were extracted using raw reads of Bm IND1 aligned to *B. melitensis* ATCC 23457 reference genome with the help of Bowtie2 and Samtools. SnpEFF was used to detect the occurrence indels and their impact on the genes.

## Figures and Tables

**Table 1:** Genome assembly statistics for Bm IND1 genome.

	All scaffolds
Number of scaffolds	29
N50 (scaffolds)	298927 bp
Longest scaffold (pseudo molecule)	609256 bp
Smallest scaffold	599 bp
Number of contigs	102
N50 (contigs)	64911 bp
Longest contig	151060 bp
Smallest contig	384 bp
Length of Chromosome I	2128440 bp
Length of Chromosome II	1185949 bp

**Table 2:** Structural annotations of Bm IND1 genome.

Attribute	Total	Chromosome I	Chromosome II
Genome size (bp)	3314389	2128440	1185949
DNA coding region (bp)	2789706 (84.16%)	1781325 (83.69%)	1008381 (85.20%)
DNA G+C content (bp)	1887544 (56.94%)	1039770 (58.37%)	588706 (58.38%)
Total genes	3258	2132	1126
RNA genes	67	49	18
Protein coding genes	3191	2083	1108
Hypothetical genes	629	446	183
rRNA genes	12	8	4
tRNA genes	55	41	14

**Table 3:** Prophage regions detected in *B. melitensis *isolate.

Strains	Region	Position	Length	GC%	Total proteins
Bm IND1	I	Chr1: 526311–540051	13.7 Kb	61.18	18
	II	Chr1: 1903640–1926292	22.6 Kb	58.14	14

Bm M28	I	Chr1: 598561–612325	13.7 Kb	61.18	18

Bm 16M	I	Chr1: 20541–37967	17.4 Kb	58.05	15
	II	Chr1: 1450561–1473186	22.6 Kb	51.51	33

**Table 4:** SNPs detected in other *B. melitensis* isolates and *B. abortus* 2308 with respect to Bm IND1.

Species	Synonymous SNPs	Nonsynonymous SNPs	Total number of SNPs
*B. melitensis* 16M	603	1462	2561
*B. melitensis* M28	56	142	281
*B. melitensis* M5-90	55	141	308
*B. melitensis* NI	78	180	351
*B. melitensis* ATCC 23457	51	124	252
*B. melitensis* ether	649	1551	2726
*B. abortus 2308*	1458	3454	6049

**Table 5:** Distribution of shared SNPs among 7 different strains of *B. melitensis *identified against Bm IND1.

Names of species sharing the SNPs in canonical manner	Number of SNPs
Bm 16M, Bm M28, Bm M5-90, Bm NI, *B. abortus*, Bm ATCC-23457, Bm Ether	141
Bm 16M, Bm M28, Bm M5-90*, B. abortus*, Bm ATCC-23457, Bm Ether	1
Bm 16M, Bm M28, Bm M5-90, Bm NI, Bm ATCC-23457, Bm Ether	1
Bm 16M, Bm M28, Bm M5-90, *B. abortus*, Bm Ether	1
*B. abortus*, Bm ATCC-23457, Bm Ether, Bm M28, Bm M5-90	4
Bm 16M, *B. abortus*, Bm ATCC-23457, Bm Ether	2
Bm 16M, *B. abortus*, Bm Ether	952
Bm 16M, B. abortus, Bm NI	1
Bm 16M, Bm M28, Bm M5-90	1
Bm 16M, *B. abortus*	16
Bm 16M, Bm ATCC-23457	1
Bm 16M, Bm Ether	16
Bm 16M, Bm M28	2
*B. abortus*, Bm Ether	66
*B. abortus*, Bm NI	1
Bm Ether, Bm NI	2
Bm M28, Bm M5-90	128
Bm 16M	1426
*B. abortus*	4864
Bm ATCC-23457	102
Bm Ether	1540
Bm M5-90	31
Bm M28	2
Bm NI	205

Bm denoted as *Brucella melitensis*.

**Table 6:** INDELs identified in Bm IND1 with respect to the *B. melitensis* ATCC 23457 in the coding region.

S. no	Gene ID	ATCC_23457	Bm IND1	Gene name
1	BMEA_RS00885	GTTTCGCGGAAGCTTTCGCGGAA	GTTTCGCGGAA	Methionine synthase
2	BMEA_RS00935	CGGG	CGGGG	Glycerol-3-phosphate dehydrogenase
3	BMEA_RS00980	GCC	GCCC	ABC transporter permease
4	BMEA_RS01770	ATTTTTT	ATTTTT	FAD-binding molybdopterin dehydrogenase
5	BMEA_RS02325	ATAT	ATATTAT	4-Hydroxy-3-methylbut-2-enyl diphosphate reductase
6	BMEA_RS03470	TGGGGGG	TGGGGGGG	Hypothetical protein
7	BMEA_RS04970	GAAAAAAA	GAAAAAAAA	Mechanosensitive ion channel protein MscS
8	BMEA_RS06240	ACCAGAAGCCCGGCCAGAAGCCCGGC	ACCAGAAGCCCGGC	Cobalt transporter
9	BMEA_RS07030	GCC	G	Aspartyl-tRNA amidotransferase subunit B
10	BMEA_RS07320	GCGCGGCGGCTCTCGCGGCGGCTCT	GCGCGGCGGCTCT	Multidrug ABC transporter ATP-binding protein
11	BMEA_RS07865	AGCGGGTAAACGGGCGGGTAAACGGGC	AGCGGGTAAACGGGCGGGTAAACGGGCGGGTAAACGGGC	Sensor histidine kinase
12	BMEA_RS08810	TCC	TC	Transporter
13	BMEA_RS10105	GT	G	Alanine acetyltransferase
14	BMEA_RS10390	TGCGTC	TGCGTCACCTTTCATGGCGTC	Hypothetical protein
15	BMEA_RS10570	TGGTGGCGGGGGTGGCGGGGGTGGCGGGGGTGGC	TGGTGGCGGGGGTGGCGGGGGTGGC	Type IV secretion protein VirB10
16	BMEA_RS12000	GCGCGTTCATCGCCGCG	GCGCGTTCATCGCCGCGTTCATCGCCGCG	Branched-chain amino acid ABC transporter ATP-binding protein
17	BMEA_RS13145	TGGGGG	TGGGGGG	Hypothetical protein
18	BMEA_RS13295	T	TA	Aminotransferase
19	BMEA_RS13570	TCCCCCCCCCC	TCCCCCCCCC	Pyridine nucleotide-disulfide oxidoreductase
20	BMEA_RS13580	AGGGGGGGGG	AGGGGGGGGGG	N-formylglutamate amidohydrolase
21	BMEA_RS13730	CTTTTTTT	CTTTTTTTT	Hypothetical protein
22	BMEA_RS14825	GAAAACGCCAAAA	GAAA	ABC transporter permease
23	BMEA_RS14895	TCCCCCCCCC	TCCCCCCCCCCCC	Amidase
24	BMEA_RS15830	CAAA	CAAAA	Amino acid ABC transporter substrate-binding protein

## Abbreviations

DIVA: Differentiating infected from vaccinated animals
MLST: Multilocus sequence typing
SNP: Single Nucleotide Polymorphism
Indels: Insertions and deletions
VNTRs: Variable Number of Tandem Repeats.
