# Supplementary material for: Genetic Characterization and Comparative Genome Analysis of Brucella melitensis Isolates from India
Source: Int J Genomics. 2016 Jul 25;2016:3034756. doi: 10.1155/2016/3034756 (PMC4976149; doi:10.1155/2016/3034756)
Supplement: Supplementary file 1 — Indels identified in the genome of Bm IND1 with respect to B. melitensis ATCC 23457. Indels were extracted using raw reads of Bm IND1 aligned to B. melitensis ATCC 23457 reference genome with the help of Bowtie2 and Samtools. SnpEFF was used to detect the occurrence indels and their impact on the genes. [file 3034756.f1.pdf]

Supplimetary Data

Supplementary Table 1. Details on sources of *Brucella melitensis* isolates and studies performed.

Sample	Source	Study
Bm IND1	Aborted goat fetus	MLST and whole genome analysis
Bm IND2	Vaginal swab of aborted goat	MLST
Bm IND3	Stomach content of aborted sheep fetus	MLST
Bm IND4	Vaginal swab of aborted sheep	MLST

Supplementary Table 2. SNPs identified using Next Generation Sequencing (NGS) data of Bm IND1 against completely sequenced B. melitensis strains. Only well-defined and characterized genome sequences of B. melitensis strains were used for the purpose of selecting closely related reference genome for Bm IND1.

**B. melitensis strains**	**Number of SNPs**
Brucella melitensis 16M	2976
Brucella melitensis M28	330
Brucella melitensis M5-90	442
Brucella melitensis NI	474
Brucella melitensis ATCC 23457	280
Brucella melitensis ether	3000
Brucella abortus 2308	6588

Supplementary Table 3. Repertoire of protein coding genes in different strains of *B. melitensis* and one *B. abortus* strain.

S. No	Organism	GenBank Assembly ID	Number of gene
1	Brucella melitensis bv. 1 str. 16M	GCA_000007125.1	3198
2	Brucella melitensis ATCC 23457	GCA_000022625.1	3136
3	Brucella melitensis bv. 1 str. Rev.1	GCA_000158695.1	3220
4	Brucella melitensis bv. 3 str. Ether	GCA_000158735.1	3237
5	Brucella melitensis bv. 1 str. 16M (1)	GCA_000160295.1	3083
6	Brucella melitensis bv. 2 str. 63/9	GCA_000182235.1	3207
7	Brucella melitensis M28	GCA_000192725.1	3363
8	Brucella melitensis M5-90	GCA_000192885.1	3360
9	Brucella melitensis NI	GCA_000227645.1	3229
10	Brucella melitensis F15/06-7	GCA_000365845.1	3154
11	Brucella melitensis B115	GCA_000365865.1	3144
12	Brucella melitensis UK22/06	GCA_000366585.1	3091
13	Brucella melitensis CNGB 1076	GCA_000366625.1	3140
14	Brucella melitensis 64/150	GCA_000366845.1	3147
15	Brucella melitensis 66/59	GCA_000366865.1	3143
16	Brucella melitensis CNGB 1120	GCA_000366885.1	3143
17	Brucella melitensis CNGB 290	GCA_000366905.1	3138

18	Brucella melitensis F10/05-2	GCA_000366925.1	3167
19	Brucella melitensis F2/06-6	GCA_000366945.1	3147
20	Brucella melitensis F3/02	GCA_000366965.1	3139
21	Brucella melitensis F5/07-239A	GCA_000366985.1	3153
22	Brucella melitensis F6/05-6	GCA_000367005.1	3140
23	Brucella melitensis R3/07-2	GCA_000367025.1	3152
24	Brucella melitensis UK19/04	GCA_000367045.1	3144
25	Brucella melitensis BG2 (S27)	GCA_000370625.1	3139
26	Brucella melitensis F1/06 B10	GCA_000370645.1	3146
27	Brucella melitensis F10/06-16	GCA_000370665.1	3143
28	Brucella melitensis F8/01-155	GCA_000370685.1	3147
29	Brucella melitensis F9/05	GCA_000370705.1	3144
30	Brucella melitensis UK14/06	GCA_000370725.1	3144
31	Brucella melitensis UK22/04	GCA_000370745.1	3158
32	Brucella melitensis UK23/06	GCA_000370765.1	3137
33	Brucella melitensis Uk24/06	GCA_000370785.1	3147
34	Brucella melitensis UK29/05	GCA_000370805.1	3156
35	Brucella melitensis UK3/06	GCA_000370825.1	3152
36	Brucella melitensis UK31/99	GCA_000370845.1	3164
37	Brucella melitensis UK37/05	GCA_000370865.1	3147

38	Brucella melitensis 02-7258	GCA_000479975.1	3151
39	Brucella melitensis 02-5863-1	GCA_000480095.1	3151
40	Brucella melitensis 11-1823-3434	GCA_000480215.1	3156
41	Brucella melitensis bv. 3 str. Ether (1)	GCA_000740355.1	3090
42	Brucella melitensis bv. 2 str. 63/9	GCA_000740395.1	3118
43	Brucella melitensis bv. 1 str. 16M (2)	GCA_000740415.1	3110
44	Brucella melitensis Human/CT/US/1995	GCA_000988815.1	3024
45	Brucella melitensis Bme20236	GCA_001431745.1	3155
46	Brucella melitensis BRUC048	GCA_001608355.1	2994
47	Brucella melitensis BRUC101	GCA_001608425.1	3000
48	Brucella melitensis bv. 3 IND1	In this study	3313
49	Brucella melitensis biovar Abortus 2308	GCA_000054005.1	3034

Supplementary Table 4. VNTRs detected in the genome of Bm IND1 and their comparison with other *B.melitensis* strains.

		Copy number				
**ID**	**Locus**	**BmIND1**	**Bm 28**	**Bm ADMAS-G1**	**Consensus Size**	**Consensus Sequence**
**VNTR-01**	chr1:1036719	2.5	2.5	2.5	11	CAGCGACAGGC
**VNTR-02**	chr1:105775	2.1	2.1	NA	19	CGCTTCCTTCAACCGCGAT
**VNTR-03**	chr1:107329	2.8	2.8	NA	12	ATGAAACCCGCA
**VNTR-04**	chr1:1085260	3.3	3.3	3.3	15	TTGAAGTTGCCAATA
**VNTR-05**	chr1:1085272	2.8	2.8	2.8	15	ATATTGAAATTGCCA
**VNTR-06**	chr1:1141914	1.9	1.9	NA	16	CGCTGGCGCTTTCATG
**VNTR-07**	chr1:1142833	2	2	NA	21	GTATGGTAAGTGACGCAAGCT
**VNTR-08**	chr1:1184601	2.8	2.8	NA	18	GAACAGAATGATTGGCCA
**VNTR-09**	chr1:1185243	2.2	2.2	NA	18	GATGAAGATTTCTTCAAC
**VNTR-10**	chr1:1206886	2.4	2.4	NA	18	AAGAAGGCGGCGCCGAAG
**VNTR-11**	chr1:1221963	2	2	NA	14	GCGGATAGTTTTTC
**VNTR-12**	chr1:1260282	1.9	1.9	1.9	15	GCCCCTGTGATTTTC
**VNTR-13**	chr1:1283476	2.4	3.4	NA	12	GGCAACAGCAAG
**VNTR-14**	chr1:1289514	2	2	NA	13	GCATTGCTTCGGG
**VNTR-15**	chr1:1322149	2.1	2.1	2.1	15	GGCGCTGCTGATGGC
**VNTR-16**	chr1:134832	2.9	2.9	NA	15	CGGAGGACATGGTGG
**VNTR-17**	chr1:147602	1.9	1.9	NA	18	GACCATCATCACGACCAT
**VNTR-18**	chr1:1575849	1.9	1.9	NA	20	TAGAGCATTTCCAGCAAATC
**VNTR-19**	chr1:1632045	2	2	NA	15	GGCGATCACAAGGGC
**VNTR-20**	chr1:164304	3.3	3.3	NA	12	TGATCGTGATGA
**VNTR-21**	chr1:1676829	2	2	NA	15	GCGTCTGGGTTTCGG
**VNTR-22**	chr1:1733517	1.9	1.9	NA	16	TCCCCTACTCCCTTAT
**VNTR-23**	chr1:1733551	4.2	4.2	NA	8	TACTGCCT
**VNTR-24**	chr1:1733554	2.1	2.1	NA	16	TGCCTTACTGCCCTAT
**VNTR-25**	chr1:1763142	2.8	2.8	NA	13	TGGCTAACCTTTC

**VNTR-26**	chr1:1942686	3.2	2.2	NA	12	GCGGGTAAACGG
**VNTR-27**	chr1:2031740	2.6	2.6	2.6	15	CGATTCTGGTTGCCA
**VNTR-28**	chr1:209539	2	2	NA	14	GAATTGCAAATGGC
**VNTR-29**	chr1:2104903	2	NA	2	15	CCCACCGGACTTGGC
**VNTR-30**	chr1:349456	3.2	3.2	NA	12	GGCGGGAGAAAG
**VNTR-31**	chr1:356129	1.9	1.9	NA	18	TTTTGAAAGGGTTTCGCC
**VNTR-32**	chr1:374624	2	NA	NA	29	TAAGGCAATAGGGCAATAGGGCAGTATGT
**VNTR-33**	chr1:374718	4.1	9.1	NA	8	ACTGCCCT
**VNTR-34**	chr1:376516	1.9	1.9	NA	29	TTAAGGCAATAGGGCAATAAGGCAGTATG
**VNTR-35**	chr1:376546	1.9	1.9	1.9	16	TAAGGGAATAGGGGAA
**VNTR-36**	chr1:376618	10.4	8.4	NA	8	ACTGCCCT
**VNTR-37**	chr1:405288	1.9	1.9	NA	18	TCGCGCTTGCCGCTTCCA
**VNTR-38**	chr1:48954	2.2	2.2	NA	15	CCCATGCCGGTCACG
**VNTR-39**	chr1:509677	2.4	2.4	NA	17	GCGCAGCCTTGCCCGCC
**VNTR-40**	chr1:589765	2.8	2.8	NA	24	AAGGATGGCAGAAAATGCCCGACC
**VNTR-41**	chr1:605931	1.9	1.9	NA	18	CCGTAGCTGCCGCTTCCC
**VNTR-42**	chr1:639735	2	2	NA	24	TTGTTGAAGGCGCTTGCCAGGGCA
**VNTR-43**	chr1:671856	2	2	NA	22	GTTTGCCCCTGCTTGCGGGTTT
**VNTR-44**	chr1:762258	3.2	3.2	NA	30	GGCCAGACTGGCCGCGTCTGAAAATAATGG
**VNTR-45**	chr1:771875	1.9	1.9	NA	16	TAAGGGAATAGGGGAA
**VNTR-46**	chr1:771946	5.5	6.5	NA	8	TACTGCCC
**VNTR-47**	chr1:778994	2	2	NA	20	TACCGCGCGAGGCGGCCCGT
**VNTR-48**	chr1:799043	2	2	NA	15	CTTGCTGGTTCTGGG
**VNTR-49**	chr1:828925	2	2	NA	21	AGGAAGCGAAAGCGCGCTTGC
**VNTR-50**	chr1:85345	3	3	NA	13	GCAGAGTTAGTGG
**VNTR-51**	chr1:881282	2.2	2.2	2.2	16	GTAAGGCAATAAGGCA
**VNTR-52**	chr1:891503	3.8	3.8	3.8	8	AGGGGAAT
**VNTR-53**	chr1:949475	1.9	1.9	1.9	16	CATGGAACAGCAGCGC
**VNTR-54**	chr2:1007937	2.1	2.1	2.1	17	ACACAGGGCCGGTACAA

**VNTR-55**	chr2:1036911	2.7	NA	NA	12	GCCTTGGGGGGG
**VNTR-56**	chr2:1045666	11	7	NA	8	GGGCAGTG
**VNTR-57**	chr2:1045821	4.4	4.4	NA	8	TACTGCCT
**VNTR-58**	chr2:1050460	2.9	2.9	NA	17	GATCATCATCACCATGG
**VNTR-59**	chr2:1050463	2.2	2.2	NA	15	CATCATCACCATGGG
**VNTR-60**	chr2:1050493	2.6	2.6	NA	15	CATGACCATCACCAT
**VNTR-61**	chr2:1051972	6	9	NA	8	GGGCAGTG
**VNTR-62**	chr2:1052060	1.9	1.9	NA	16	TCCCCTACTCCCTTAT
**VNTR-63**	chr2:1086220	2.1	2.1	2.1	18	GCAGGCAGCACAGAACAG
**VNTR-64**	chr2:1089799	2.5	2.5	2.5	28	GCACGCTCATCGTGCCGTAATAAAAGAA
**VNTR-65**	chr2:115304	3.8	3.8	NA	15	TTCATGGCGTCGCCT
**VNTR-66**	chr2:164963	2.1	2.1	NA	12	CGATCTTGCCGT
**VNTR-67**	chr2:170184	8.1	6.1	NA	8	ATTCCCCT
**VNTR-68**	chr2:217770	1.9	1.9	NA	18	GCAATGACCAGAAAGGAA
**VNTR-69**	chr2:290120	2	2	NA	20	GAAGCAGAAAGCGCCAGCAA
**VNTR-70**	chr2:330625	2.2	2.2	NA	21	TGCTGAAATAGCCGATGACCA
**VNTR-71**	chr2:453544	3.1	NA	NA	12	GTTCATCGCCGC
**VNTR-72**	chr2:667985	2	2	2	22	CGATATCGAGCGCACCAACGGT
**VNTR-73**	chr2:711337	1.9	1.9	1.9	13	TGTGAAGATGCGA
**VNTR-74**	chr2:826002	5.4	4.4	NA	8	TAAGGGAG
**VNTR-75**	chr2:832157	2.3	3.3	2.3	12	GGCATCATGGCG
**VNTR-76**	chr2:93813	2	2	NA	20	ATCAACTATCTGCGCGAAAG
**VNTR-77**	chr2:93819	2.3	2.3	NA	20	TATCTGCGCGAAAGACCAAC
**VNTR-78**	chr2:958168	2	2	2	21	TCGGATTGGTTTATCAAAATG

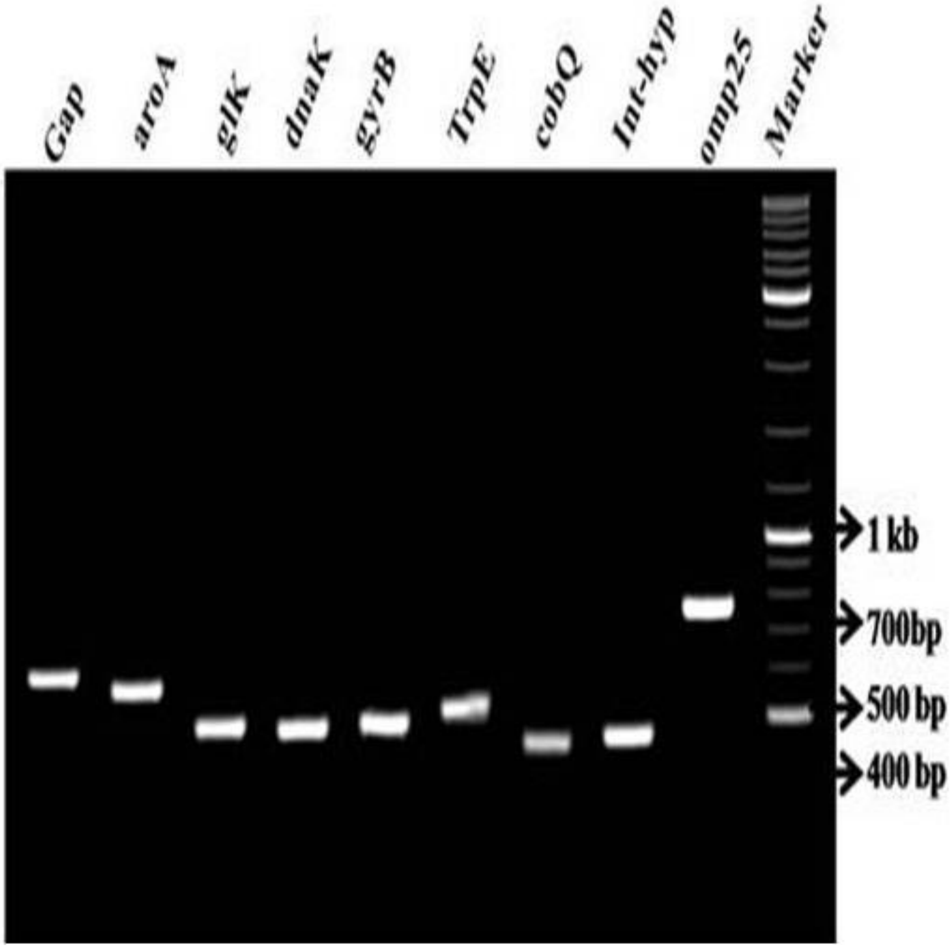

Supplementary Figure 1. Gel photo showing the amplification of nine loci from *B. melitensis* IND1 strain. Amplification of nine loci from *B. melitensis* IND1 strain for MLST analysis.

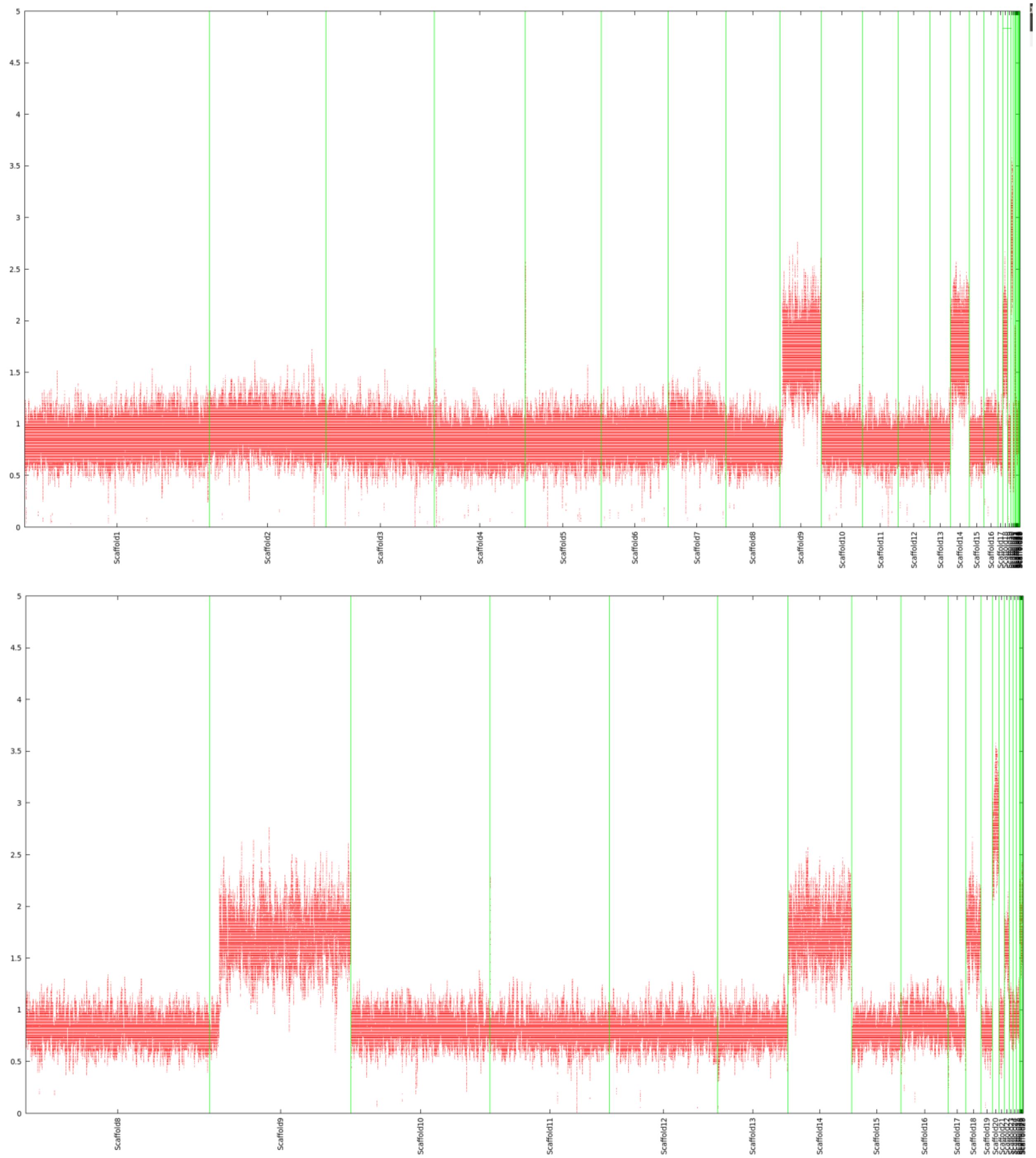

Supplementary Figure 2. The depth coverage of each scaffold of Bm IND1 genome. (a) X-axis shows the relative length of scaffolds and Y axis represents each position covered with number of reads on a scale of 100. Chart was generated using GNUplot. Scaffold 9, scaffold 14 and scaffold 18 are showing higher coverage. Scaffold 20 was showing maximum coverage. (b) Re-plot of the data (A) with X-axis shifted right to highlight smaller scaffolds.

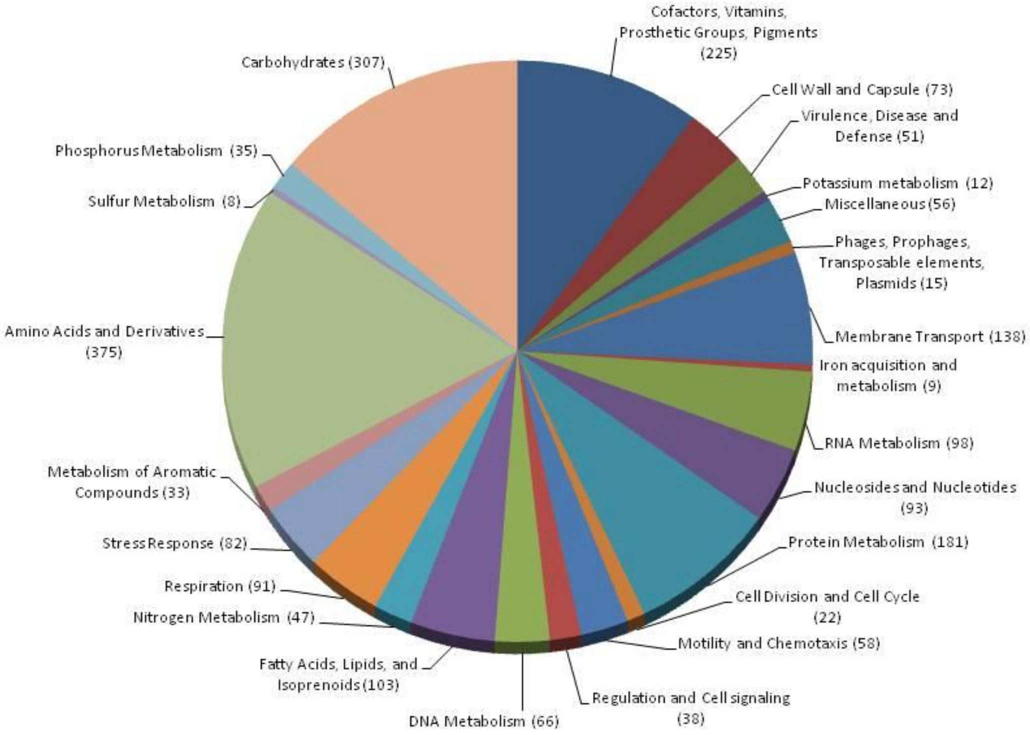

Supplementary Figure 3. The distribution of genes containing SNPs in *B. melitensis* IND1 against other *B. melitensis* strains. *B. melitensis* IND1 genes carrying SNPs with respect to any of the seven *B. melitensis* strains (*B. melitensis* 16M, *B. melitensis* Ether, *B. melitensis* NI, *B. melitensis* M5-90, *B. melitensis* M28, *B. melitensis* ATCC 23457) *or B. abortus* 2308 are organized as per SEED sub-system annotation
